# Radiochemical examination of transthyretin (TTR) brain penetration assisted by iododiflunisal, a TTR tetramer stabilizer and a new candidate drug for AD

**DOI:** 10.1038/s41598-019-50071-w

**Published:** 2019-09-20

**Authors:** Xabier Rios, Vanessa Gómez-Vallejo, Abraham Martín, Unai Cossío, Miguel Ángel Morcillo, Mobina Alemi, Isabel Cardoso, Jordi Quintana, Jesús Jiménez-Barbero, Ellen Y. Cotrina, Gregorio Valencia, Gemma Arsequell, Jordi Llop

**Affiliations:** 1 Radiochemistry and Nuclear Imaging Group, CIC biomaGUNE, 20014 San Sebastián, Guipúzcoa Spain; 2 Achucarro Basque Center for Neuroscience, 48940 Leioa, Spain; 3 Ikerbasque Basque Foundation for Science, Maria Díaz de Haro 3, 48013 Bilbao, Spain; 4 Biomedical Applications of Radioisotopes and Pharmacokinetics Unit, CIEMAT, 28040 Madrid, Spain; 5 IBMC - Instituto de Biologia Celular e Molecular, i3S-Instituto de Investigação e Inovação em Saúde, 4200-135 Porto, Portugal; 6 Plataforma Drug Discovery, Parc Científic de Barcelona (PCB), 08028 Barcelona, Spain; 7 Present Address: Research Programme on Biomedical Informatics, Universitat Pompeu Fabra, 08003 Barcelona, Spain; 8 CIC bioGUNE, Bizkaia Technology Park, Building 800, 48160 Derio, Spain; 9 Institut de Química Avançada de Catalunya (I.Q.A.C.-C.S.I.C.), 08034 Barcelona, Spain; 10 Centro de Investigación Biomédica en Red- Enfermedades Respiratorias (CIBERES), Madrid, Spain

**Keywords:** Proteins, Proteins, Blood-brain barrier, Blood-brain barrier

## Abstract

It is well settled that the amyloidogenic properties of the plasma protein transporter transthyretin (TTR) can be modulated by compounds that stabilize its native tetrameric conformation. TTR is also present in cerebrospinal fluid where it can bind to Aβ-peptides and prevent Aβ aggregation. We have previously shown that treatment of Alzheimer’s Disease (AD) model mice with iododiflunisal (IDIF), a TTR tetramer stabilizing compound, prevents AD pathologies. This evidence positioned IDIF as a new lead drug for AD. In dissecting the mechanism of action of IDIF, we disclose here different labeling strategies for the preparation of ^131^I-labeled IDIF and ^131^I- and ^124^I-labeled TTR, which have been further used for the preparation of IDIF-TTR complexes labeled either on the compound or the protein. The biodistribution of all labeled species after intravenous administration has been investigated in mice using *ex vivo* and *in vivo* techniques. Our results confirm the capacity of TTR to cross the blood brain barrier (BBB) and suggest that the formation of TTR-IDIF complexes enhances BBB permeability of both IDIF and TTR. The increased TTR and IDIF brain concentrations may result in higher Aβ-peptide sequestration capacity with the subsequent inhibition of AD symptoms as we have previously observed in mice.

## Introduction

After more than a hundred years of knowing and dealing with the disease, the crude reality is that medicinal chemistry can offer little wonder to Alzheimer’s Disease (AD) patients. Even worst, most recent efforts at AD therapies are failing at advanced Phase III clinical trials after a great deal of expenditure and human suffering^1–5^. Most efforts towards developing AD pharmacological therapies have been focusing into the burden posed by the excess of Aβ-peptides in the brain, which can aggregate producing toxic oligomers and plaques. The recent investigations to understand and exploit protein and Aβ-peptides interactions are a shift from these traditional strategies^6^. These studies target the same Aβ problem, but from the perspective of preventing Aβ aggregation by protein-Aβ complex formation, which stabilizes soluble Aβ-peptides or facilitates their efflux from the brain. Some of these proteins are the carrier proteins ApoJ (clusterin)^7^ and ApoE^8^, human serum albumin^9,10^ and transthyretin (TTR)^11–13^.

TTR, a 55 kDa homotetramer^14^ with a dimer of dimers quaternary structure, is a protein present in the serum and cerebrospinal fluid^15^ (CSF) that carries retinol and the thyroid hormone thyroxine (T4)^16^. The stability of the tetrameric form of TTR plays a pivotal role in the amyloidogenic properties of the native protein and its mutants^17,18^, which are in the cause of a series of severe amyloid diseases, *i.e.* Familial Amyloidotic Polyneuropathy^19^ and Familial Amyloid Cardiomyopathy^20^. Owing that tetramer dissociation into monomers is believed to be the first step leading to TTR amyloid formation^21^, a number of small molecule compounds that bind and stabilize the tetramer have been developed as drugs for arresting these amyloid diseases^22–24^. One of them, tafamidis (Vyndaqel), has reached the status of European Medicines Agency (EMA) and Japan approved orphan drug^25,26^. Another stabiliser is the drug Tolcapone, approved for the treatment of Parkinson’s disease, and being repurposed for the treatment of TTR-related amyloidosis^27^.

Evidence has also been mounting on the involvement of TTR in AD pathology. For instance, TTR concentration in CSF of AD patients is lower than that in healthy subjects, turning TTR into a disease marker for AD^28,29^. The affinity of TTR for Aβ-peptides is also well known, although only recently it has been considered as a possible AD disease target^13,30,31^. In this concern, our group has been working on the hypothesis that a key factor for TTR and Aβ-peptides affinity and subsequent Aβ amyloid inhibition is TTR tetramer stability^29,32,33^. An efficient way to enhance TTR tetramer stability is to generate TTR-stabilizer complexes. To investigate if these mechanisms may occur *in vivo*, in our previous work we dosed AD transgenic mice with our proprietary TTR stabilizer compound, iododiflunisal (IDIF)^34–36^. We observed a marked decrease in brain Aβ-peptides levels and deposition as well as improved cognitive function associated with the AD-like neuropathology in these animals^37^. In this previous work, animals started treatment before the beginning of amyloid deposition, suggesting that IDIF administration prevented Abeta deposition. Another work showed that TTR assists Aβ brain efflux at the BBB and also its uptake by the liver, probably through low density lipoprotein receptor-related protein 1 (LRP1)^38^; authors also showed that TTR stability, which is impaired in AD^29,39^, is important in such TTR-assisted Aβ transport and can be enhanced by TTR stabilizers, namely IDIF^39^. Taken together, these results suggest that TTR, by binding Aβ peptide, participates in its transport from the brain to the periphery and in its subsequent uptake and degradation at the liver, thus preventing Aβ brain deposition and avoiding AD progression.

In the current work, we have tackled a radiochemistry-based study with the ultimate goal of shedding light on BBB^40–42^ penetration capacity of IDIF and TTR either as free compounds or in the form of a TTR-IDIF complex. With that aim, we first developed methods for the radioiodination^43^ of TTR both using Iodine-131 (^131^I) and Iodine-124 (^124^I), and investigated the biodistribution pattern and basic pharmacokinetic properties after intravenous administration in wild type mice using dissection/gamma counting and *in vivo* Positron Emission Tomography (PET) imaging^44^. The capacity to cross the BBB and the regional distribution within the brain were also investigated using autoradiography. In order to elucidate the effect of the stabilizer, parallel biodistribution studies were carried out with the IDIF-stabilized protein (TTR-IDIF complex), by incorporation of the radiolabel in the TTR ([^131^I]TTR-IDIF) or in the stabilizer (TTR-[^131^I]IDIF). Finally, the biodistribution of the free small-molecule stabilizer (IDIF) was also investigated.

## Results and Discussion

### Radiochemistry: Synthesis of [^131^I]TTR, [^124^I]TTR and [^131^I]IDIF

In spite of the important biological roles of TTR and the fact that one of the TTR tetramers stabilizers (tafamidis) has entered clinical practice, very little is known about the pharmacokinetic properties of this protein, its capacity to cross the BBB and how stabilizers alter such pharmacokinetics. One previous study reporting the distribution of TTR at the whole body level has demonstrated that intravenously administered recombinant wild-type TTR (rTTR) presents a plasma concentration curve with an elimination-phase half-life (t_1/2β_) of 2.53 ± 0.26 h in wild type rats^45^. The protein was mainly accumulated in the kidneys, liver, stomach and small intestine at 4 hours after administration. Unfortunately, no data about the presence of the labeled compound in the brain was reported. Pharmacokinetic studies to investigate the behaviour of different TTR binding thyroid hormones like T4 and TTR tetramer stabilizers is also scarce. In one of the examples, the transport of ^125^I-labeled T4 from the CSF into brain and choroid plexus (CP) was measured in anesthetized rabbits^46^. The authors observed a carrier mediated distribution of T4 from the CSF into the brain and CP, which was TTR dependent. In a more recent work, in one of our laboratories we have used mass spectroscopy techniques to show that IDIF administered orally to mice was able to reach the cerebrospinal fluid, while TTR levels both in plasma and cerebrospinal fluid remained unaltered^37^.

In order to examine their ability to cross the BBB, we have revisited the pharmacokinetic properties of both TTR and IDIF. Radioiodination has been selected as the best labeling strategy because: (i) in the case of IDIF, no structural changes are introduced in the molecule; and (ii) in the case of TTR, radioiodination is the most frequent labelling techniques for proteins.

The radiolabeling of TTR was carried out *via* electrophilic aromatic iodination on the tyrosine residues by incubation of the protein with Na[^124^I]I or Na[^131^I]I in the presence of an oxidizing agent, following a well-established method widely described in the literature^47^. In our hands, decay-corrected radiochemical yields ca. 50% for ^131^I and 14% for ^124^I could be achieved. The lower radiochemical yields obtained with ^124^I were expected, and these could have been increased by adding carrier NaI as previously reported in the literature^48^, at the cost of introducing more iodine atoms per protein, which may compromise or alter the biological function. In the case of TTR these effects are particularly crucial since small changes such as one point mutation as mild as a Phe for Tyr (Y78F TTR) results in a protein that shows very different properties. Hence, and considering that the yields were sufficient to approach subsequent studies, we decided to use carrier-free ^124^I. Both [^131^I]TTR and [^124^I]TTR, which were obtained as tetramers as observed by Native-PAGE analysis, showed excellent radiochemical purity, exceeding 98% at the end of the synthesis (EoS).

For the radiolabelling of IDIF, a similar protocol was used. However, the stronger oxidizing agent Chloramine-T was used in this case^49,50^. By careful optimization of the reaction conditions (temperature, pH and reaction time) decay-corrected radiochemical yields close to 20% could be achieved, with the radiochemical purity of the labeled compound exceeding 95% at the EoS.

### Preparation of TTR-[^131^I]IDIF and [^131^I]TTR-IDIF complexes

As previously mentioned, one of our aims was to compare the pharmacokinetic properties of the protein and the stabilizer when administered alone or in complex to see if a synergic effect due to TTR tetramer stabilization was at play. Hence, we decided to prepare two complexes, one labeled on the protein and one on the stabilizer to see possible deleterious effects of radioiodination on this very structurally sensitive protein.

**Figure 1:**
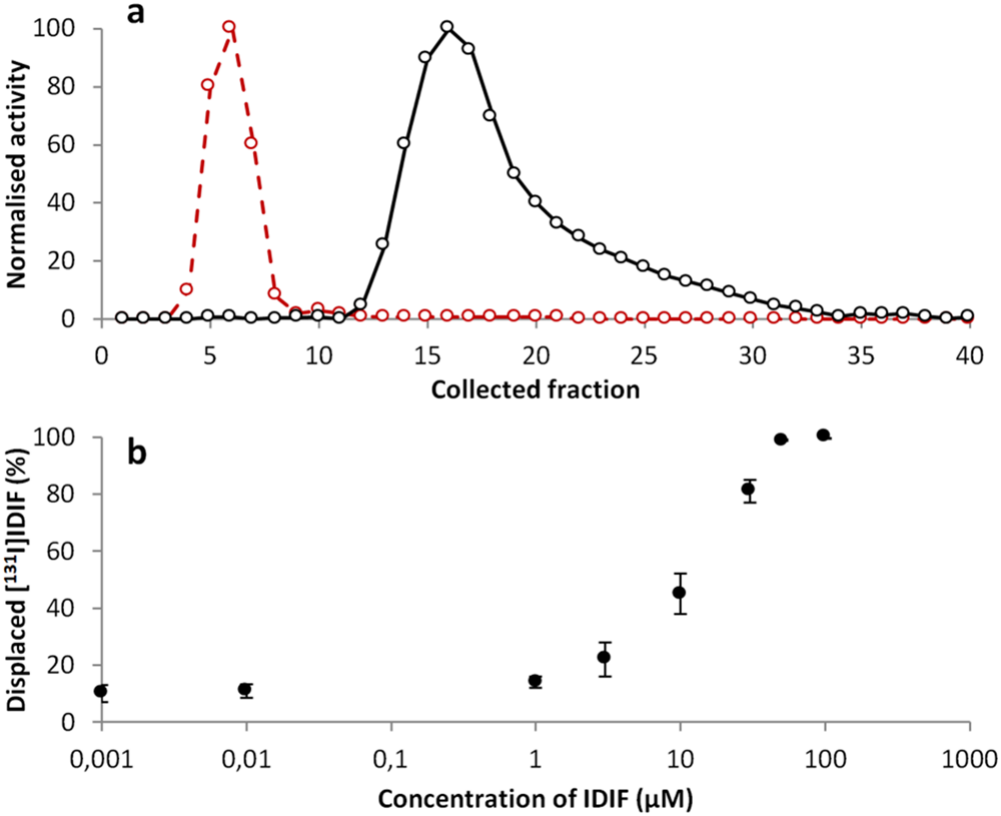
(**a**) Amount of radioactivity in the different fractions after elution of [^131^I]IDIF (black dots) and [^131^I]IDIF-TTR complex (red dots) through the Illustra NAP-5 column; (**b**) Percentage of [^131^I]IDIF displaced at different concentrations of IDIF.

The synthetic method for the preparation of TTR-[^131^I]IDIF was designed to ensure that the whole amount of [^131^I]IDIF was in the form of the complex with TTR. With that aim, the IDIF/TTR molar ratio was kept to a minimum. This was achieved by using carrier free [^131^I]IDIF. After incubation for the formation of the complex, the purification was achieved by size exclusion chromatography. After elution, the amount of radioactivity in the different collected fractions (ca. 100 µL each) revealed that all the [^131^I]IDIF was in the form of the complex (see Fig. 1a for example of elution profiles corresponding to “free” [^131^I]IDIF and TTR-[^131^I]IDIF). As it can be seen, both fractions could be efficiently resolved and pure TTR-[^131^I]IDIF could be obtained by combining fractions 4–6.

Likewise, the preparation of [^131^I]TTR-IDIF was carried out under adequate conditions to ensure that all the [^131^I]TTR was stabilized with IDIF. This is extremely important because the separation of [^131^I]TTR from [^131^I]TTR-IDIF is unfeasible using chromatographic techniques. Hence, the required concentration of IDIF to fully occupy the binding sites of all [^131^I]TTR molecules was determined in a first step by incubating TTR-[^131^I]IDIF with increasing concentrations of non-labeled IDIF (see Fig. 1b). It was observed that a concentration of IDIF = 50 µM displaced > 99% of bound [^131^I]IDIF. Hence, these experimental conditions were used for the preparation of [^131^I]TTR-IDIF to be used in animal experiments.

### Biodistribution studies

**Figure 2:**
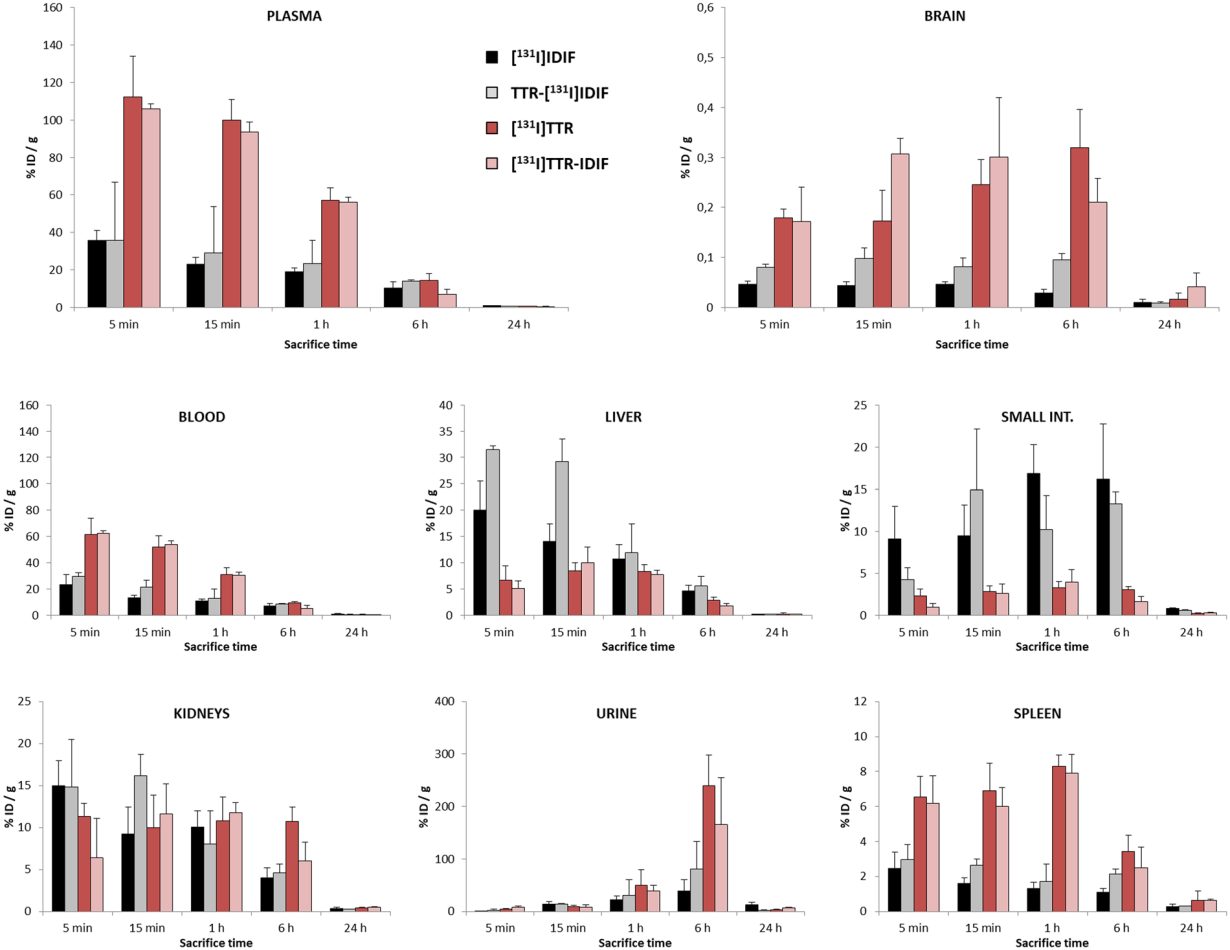
Accumulation of radioactivity in different organs and fluids obtained at different time points after intravenous administration of [^131^I]IDIF (black bars), TTR-[^131^I]IDIF (grey bars), [^131^I]TTR (red bars) and [^131^I]TTR-IDIF (pink bars). Values of percentage of injected dose (% ID) per gram of tissue are expressed as mean ± standard deviation, n = 3 per compound and time point.

The biodistribution of [^131^I]TTR after intravenous administration in wild type mice was first investigated. With that aim, animals (n = 3 per time point) were administered with the radiotracer and sacrificed by exsanguination at pre-defined time points. Major organs were harvested together with blood and urine and the amount of radioactivity was determined by gamma counting. (Fig. 2, red bars).

As it can be seen in Fig. 2, the presence of [^131^I]TTR in the blood was pronounced, with values of percentage of injected dose per gram of blood (%ID/g) of 61.4 ± 12.6 at t = 5 min. This value progressively decreased afterwards to reach levels below 1% at t = 24 h. A similar trend is observed for plasma, although higher values are reached in this fluid. The labeled protein also accumulated rapidly in the spleen (ca. 6% ID/g at t = 5 min) and in the kidneys (11.35 ± 1.57%  ID/g at t = 5 min), while the presence in the liver (6.59 ± 2.84% ID/g) and in the small intestine (2.30 ± 0.83% ID/g) was also noticeable at this time point. The presence of [^131^I]TTR in the kidneys at late time points (e.g. 10.71 ± 1.74% ID/g at t = 6 h) together with the high concentration in urine suggest preferential urinary elimination. Besides the general information regarding biodistribution, one of the most impacting results is the marked accumulation of [^131^I]TTR in the brain, which reaches values of 0.18 ± 0.02% ID/g at t = 5 min after administration and progressively increases up to 0.32 ± 0.08% ID/g at t = 6 h after administration.

We next investigated the biodistribution of the [^131^I]TTR-IDIF complex using the same experimental approach (Pink bars, Fig. 2). In general terms, very similar distribution patterns to those obtained for [^131^I]TTR were observed, with pronounced accumulation in the spleen at short time points which was hold up to 6 h after administration; presence of radioactivity in the kidneys, liver and small intestine; and progressive elimination *via* urine. Noteworthy, the accumulation of radioactivity in the brain was slightly different from that obtained for [^131^I]TTR. At short times after administration (15 min) statistically significant higher values were obtained for [^131^I]TTR-IDIF (P = 0.04). These differences disappeared at t = 1 h and statistically equivalent values for [^131^I]TTR-IDIF and [^131^I]TTR were obtained at t > 1 h. These results suggest that TTR stabilisation may have an impact on its BBB penetration capacity at short time points. However, the presence of endogenous stabilisers may lead to a progressive formation of the TTR-endogenous stabiliser complex, and this would explain why the entrance of [^131^I]TTR in the brain is delayed with respect to [^131^I]TTR-IDIF.

Lastly, we investigated the biodistribution of the “free” stabilizer ([^131^I]IDIF, black bars in Fig. 2) and the complex TTR-IDIF but incorporating the ^131^I atom on the stabilizer (grey bars in Fig. 2). The concentration of radioactivity in blood of the free stabilizer ([^131^I]IDIF) peaked at t = 5 min after administration (23.3 ± 7.6%ID/g) to progressively decrease afterwards; the concentration of radioactivity in plasma followed a similar trend, although higher values were obtained, irrespective of the time point. A fast accumulation in the liver and the kidneys was observed at short times after administration (20.0 ± 5.5 and 14.9 ± 3.0%ID/g, respectively, at t = 5 min) followed by a progressive decline, which was paralleled by a progressive accumulation of radioactivity in the small intestine which peaked at t = 1–6 h (ca. 15% ID/g) and a slow elimination *via* urine. The presence of [^131^I]IDIF in the brain was low over the whole duration of the study, with maximum values of 0.05%ID/g at 1 h after administration. The low concentration of radioactivity in the brain suggests that the formation of the TTR-[^131^I]IDIF complex does not take place *in vivo*, probably due to the presence of endogenous stabilizers that act as competitors.

The biodistribution of TTR-[^131^I]IDIF (Fig. 2, grey bars) showed very similar patterns to those obtained for “free” [^131^I]IDIF, although some differences are worth to mention. First and more obvious, the concentration of radioactivity in the brain is substantially higher at t = 5 min, 15 min, and 6 h after administration, with P values with respect to [^131^I]IDIF of 0.02, 0.0097 and 0.001, respectively. The P value at t = 1 h is 0.08. However, the general trend suggests that inclusion of 1–2 additional animals might lead to more prominent differences also at this time point. To our understanding, these results point to the role of TTR in contributing to the capacity of IDIF to penetrate the BBB. Actually, the capacity of TTR to assist T4 transport from blood to brain across the blood- CSF barrier (BCSFB) has been previously suggested^51,52^. The formation of the TTR-[^131^I]IDIF complex also contributes to a major accumulation of radioactivity in the liver, which is significant at t = 5 min (P = 0.023) and at t = 15 min (P = 0.0086). At longer time points, both compounds [^131^I]IDIF and TTR-[^131^I]IDIF show equivalent accumulation in the liver.

Direct comparison between the distribution patterns observed for TTR-[^131^I]IDIF (grey bars in Fig. 2) and [^131^I]TTR-IDIF (pink bars in Fig. 2) suggest that the complex TTR-IDIF does not remain stable over the whole duration of the experiments. If this was the case, both patterns should perfectly match. However, some differences in the distribution can be observed. Together with the fact that the patterns found for [^131^I]IDIF and TTR-[^131^I]IDIF on one hand, and for [^131^I]TTR and [^131^I]TTR-IDIF on the other hand are also not equivalent as discussed previously, this result confirms that dissociation of the TTR-IDIF complex occurs *in vivo*, although this is a progressive process. Such dissociation might be due to the displacement of the stabilizer (IDIF) by an endogenous ligand (e.g. T4), although this is just a hypothesis that remains unproved.

### Plasma clearance

To better investigate the potential effect of the stabilizer in the pharmacokinetics of TTR, we performed separated experiments to determine the plasma clearance of both [^131^I]TTR and [^131^I]TTR-IDIF. With that aim, mice (n = 4 per compound) were intravenously administered and blood samples were withdrawn at different time points, and the amount of radioactivity in each sample was determined. Values were corrected by the plasma/blood fraction and pharmacokinetic data analysis was performed on individual plasma concentrations and then averaged (Table 1).

**Table 1:** Main pharmacokinetic parameters of [^131^I]TTR and [^131^I]TTR-IDIF estimated after a single intravenous administration in mice.

Parameter	[^131^I]TTR	[^131^I]TTR-IDIF
K_el_ (h^−1^)	0.450 ± 0.082	0.465 ± 0.093
t_1/2_ (h)	1.59 ± 0.32	1.54 ± 0.30
V_d_ (mL)	1.84 ± 0.22	2.22 ± 0.29
Cl (mL/h)	0.82 ± 0.14	1.03 ± 0.26

The pharmacokinetics was best described by an open mono-compartmental model in both groups, with an elimination half-life of approximately 1.5 hours. There were no statistically significant differences between the groups for any of the pharmacokinetic parameters (P values of 0.82, 0.83, 0.082 and 0.20 for K_el_, t_1/2_, V_d_ and Cl, respectively). The similarity in the pharmacokinetics of [^131^I]TTR-IDIF and [^131^I]TTR, together with the similar biodistribution patterns obtained by dissection/gamma counting, again suggest that the complex is progressively dissociated after administration.

### TTR passage of BBB by autoradiography

**Figure 3:**
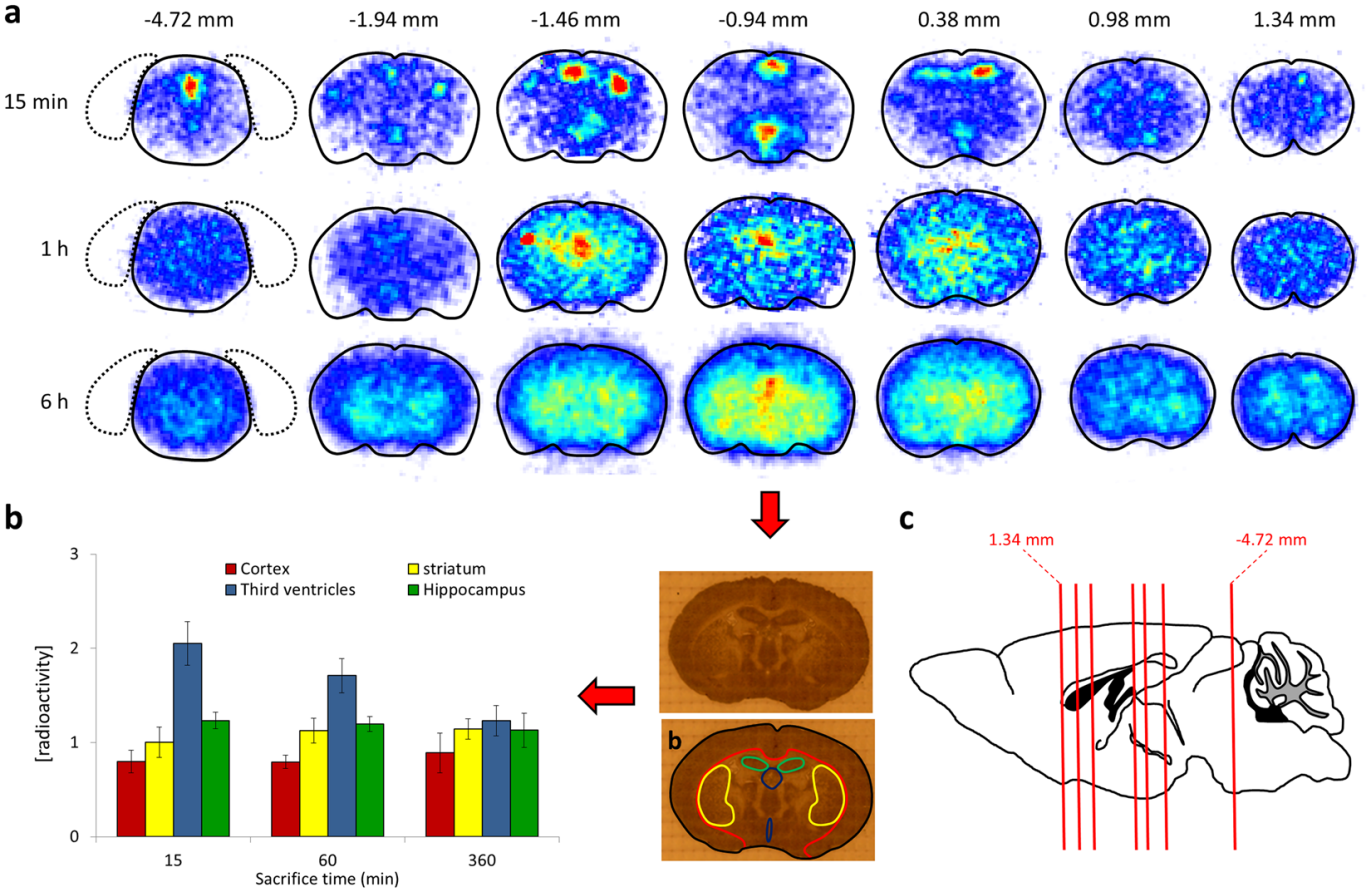
(**a**) Autoradiography studies of brain slices collected at 15 min, 1 h and 6 h after intravenous administration of [^131^I]TTR at different Bregma values. The contour of the brain is delineated in all cases for clarity; (**b**) Picture of a representative slice corresponding to Bregma = −0.94 mm. The cortex (red), hippocampus (green), striatum (yellow) and third ventricles (dark blue) are delineated; three consecutive slices containing the regions mentioned above were analyzed per animal and time point, and the results are represented as concentration of radioactivity in each region, relative to the concentration in the whole brain slice; (**c**) Schematic representation of the mouse brain (sagittal view). The positions of the different Bregma values for which autoradiography images are shown in (**a**) are indicated with red lines.

To confirm the capability of [^131^I]TTR to cross the BBB, and in order to gain information about the regional distribution of the labeled protein within the brain as a function of time, autoradiography studies were carried out on selected brain slices (Fig. 3).

The images clearly show that at short times after administration, [^131^I]TTR is concentrated in different spots: The aqueduct (Bregma = −4.72 mm), the third and lateral ventricles (Bregma = −1.94, −1.46 and −0.94 mm), and the lateral ventricles (Bregma = 0.38 mm). At 1 hour, the radioactive signal delocalizes over the whole brain, reaching regions such as the striatum, the hippocampus and the cortex, although hot spots can still be detected in the lateral and third ventricles. Finally, at t = 6 h, the radioactivity is uniformly distributed over the whole brain and no clear hot spots can be detected. These results point to the capability of TTR to enter the cerebrospinal fluid and finally the brain, leading to an almost uniform distribution within this organ.

In order to get more quantitative data, regions of interest were delineated on the photographs of selected slices (Bregma = −0.94) and translated to the autoradiography images, and the ratio between the concentration of radioactivity in the region and the concentration of radioactivity in the whole slice was determined for each brain region (Fig. 3b). Slices at Bregma = −0.94 were selected as they contain cortex, striatum, hippocampus and ventricles. As it can be seen in Fig. 3b, at short times after administration (15 min) the concentration of radioactivity in the third ventricles doubles the average concentration in the whole brain slice. At long time points (6 h) the concentration of radioactivity is almost uniform and all brain sub-regions show similar values.

### *In vivo* imaging

**Figure 4:**
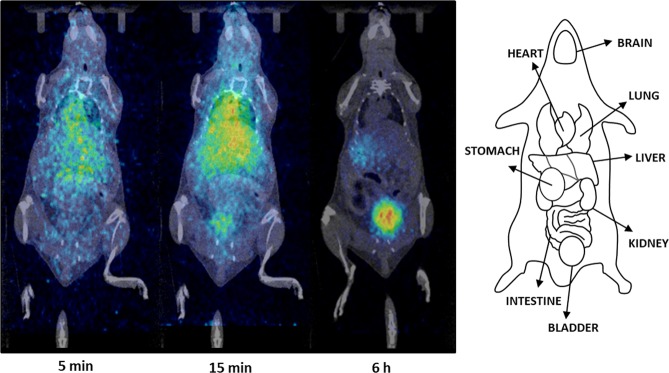
PET-CT images obtained at different time points after intravenous administration of [^124^I]TTR. Representative coronal slices have been co-registered with CT images of the same animal for better localization of the radioactive signal. On the right, major organs are schematically identified.

*Ex vivo* biodistribution studies performed by dissection and gamma counting offer accurate results about the amount of radioactivity present in the different organs. However, such results expressed by bar diagrams are less suggestive than body images. Thus, we have also considered the possibility to investigate the biodistribution of TTR using Positron Emission Tomography (PET) and [^124^I]TTR as radiotracer. In Fig. 4, representative PET-CT images acquired at t = 5 min, 15 min and 6 h after administration of the labelled protein are shown. Visual inspection of the images confirms the presence of [^124^I]TTR in the liver at early time points and elimination via urine at late time points after administration. The significant signal in the heart, especially at short times after administration, can be interpreted as a surrogate of the presence of a high concentration of radioactivity in blood, which perfectly matches with *ex vivo* findings. PET images also revealed significant accumulation of radioactivity in the stomach, which was evident at late time points when radioactivity was cleared from all major organs (see t = 6 h in Fig. 4).

Positron Emission Tomography has a limited spatial resolution, which is close to 1 mm for preclinical scanners. Additionally, elimination of the blood contribution from the radioactive signal is extremely challenging and requires some assumptions that may pose significant error in the final data. Hence, quantification of the amount of radioactivity in organs or tissues with low uptake might be subjected to large errors; accordingly we could not confirm by this technique if TTR crosses the BBB and distributes throughout the brain.

## Conclusions

The findings presented here demonstrate that the biodistribution in mice of four radiolabeled species, namely [^131^I]IDIF, TTR-[^131^I]IDIF, [^131^I]TTR and [^131^I]TTR-IDIF can be investigated using dissection and gamma counting. Our results confirm BBB permeability to IDIF and TTR. For the first time, the pivotal role of TTR as a carrier of IDIF through the BBB is revealed. It is suggested that the complex TTR-IDIF is not stable *in vivo* after intravenous administration, and progressively dissociates. Autoradiography studies confirmed the BBB crossing capacity of TTR and provided relevant information about the regional distribution of the labelled compound within the brain at different time points after administration.

## Materials and Methods

### General

[^131^I] NaI (solution in 0.1 M NaOH) and [^124^I] NaI (solution in 0.02 M NaOH) were purchased from Perkin Elmer. Ultrapure water (resistivity > 18MΩcm) was generated using a Milli-Q system (Millipore, Bedford, MA, USA). Iododiflunisal (IDIF) was prepared by iodination of the NSAID following our reported procedures^36^ and the corresponding water soluble meglumine salt was prepared following described procedures^53^. The production of recombinant human protein wt-TTR was performed following previously reported methods^54^. The NSAID Diflunisal (DIF) and other chemicals and solvents (unless otherwise specified) were of analytical grade and purchased from Sigma-Aldrich.

### Radiochemistry

Preparation of [^131^I]iododiflunisal (IDIF) was carried out by ^131^I-radioiodination of the NSAID diflunisal (DIF) using electrophilic aromatic iodination reaction. In brief, DIF (0.4 mg) was dissolved in EtOH (120 µL) in a polypropylene tube (Eppendorf® Safe-lock, 1.5 mL) previously loaded with [^131^I]NaI (3 µL of the commercially available solution, amount of radioactivity ca. 3.7 MBq). A solution of Chloramine-T (0.6 mg) in ultrapure water (80 µL) and aqueous NaOH solution (0.01 M, 100 µL) were sequentially added. The mixture was allowed to react for 20 minutes at room temperature and the crude was purified by high performance liquid chromatography (HPLC) using a Mediterranea Sea18 C18 column (150 × 4.6 mm, 5 μm particle size; Teknokroma, Spain) as the stationary phase and aqueous NaH_2_PO_4_ 0.05 M solution, pH 3.4/acetonitrile 60/40% v/v as the mobile phase (flow rate = 1 mL/min). The collected fraction (retention time = 12 min) was eluted through a C18 solid phase extraction cartridge (Sep-Pak C18 light, Waters) to selectively retain [^131^I]IDIF. The cartridge was rinsed with water (10 mL) and the labelled product was finally eluted with ethanol (500 μL). Chemical and radiochemical purities were determined by HPLC.

Radioiodination of transthyretin (TTR) using either ^131^I or ^124^I was carried out by electrophilic aromatic substitution on the tyrosine residues. An aqueous solution of TTR (4 µL, 3 µg/μL) was introduced into a Iodogen reagent (Thermo Scientific, USA) coated polypropylene tube and incubated at room temperature with the corresponding radioiodine solution (commercially available solution, amount of radioactivity 7.4–74 MBq) in the presence of phosphate buffered saline (PBS) solution (16 µL, 0.5 M, pH = 7.4). Gentle shaking was applied at 2 minute intervals for 20 min. Subsequently, 250 µL of PBS solution (0.01 M, containing NaCl 1 M, pH = 7.4) was added and the mixture was transferred to a second polypropylene tube containing Na_2_S_2_O_3_ (50 µL, 0.1 M). The reaction crude was purified using an Illustra NAP-5 column (GE Healthcare, USA) with TNE buffer (Tris 0.1 M, NaCl 0.1 M, EDTA 1 mM, pH = 8) as the mobile phase. The eluted product was collected in 150 µL fractions and those containing the labelled TTR were combined for subsequent experiments. The chemical and radiochemical purity of the labelled TTR were determined by radio-thin layer chromatography (radio-TLC) using iTLC-SG chromatography paper (Agilent Technologies, CA, USA) and ethanol/water (85/15 v/v) as the mobile phase. Native-PAGE analyses were carried out on an omniPAGE Mini system (Omnilab-Laborzentrum GmbH & Co. KG, Bremen, Germany) at constant voltage of 180 V for 50 min to assess the size of the protein after reaction. The reading was performed with a TLC-reader (MiniGITA, Raytest).

### Preparation of TTR-[^131^I]IDIF complex

A solution of TTR (4 µL, 3 μg/μL), 50 µL of [^131^I]IDIF (1.8 MBq) and 946 µL of TNE buffer were mixed (final concentration of TTR = 0.22 μM) and incubated at 4 °C overnight. A fraction of the mixture (100 µL) was purified using an Illustra^TM^ NAP-5 column (GE Helathcare, USA) with TNE buffer (Tris 0.1 M, NaCl 0.1 M, EDTA 1 mM, pH = 8) as the mobile phase. Different fractions containing 100 μL were collected and the amount of radioactivity was measured in a dose calibrator (Carpintec CRC-25, USA). Those fractions containing the TTR-[^131^I]IDIF complex were combined to approach subsequent experiments.

### Preparation of [^131^I]TTR-IDIF complex

First, the TTR/IDIF relative amount to achieve complete formation of the complex was determined. With that aim, six 100 μL aliquots of the [^131^I]IDIF-TTR complex prepared as described above were incubated overnight (T = 4 °C) with different amounts of non-radioactive IDIF in 100 μL TNE buffer (final concentrations of IDIF = 0.88, 2.63, 8.76, 17.5, 52.5 and 175 μM, respectively; final concentration of TTR as TTR-[^131^I]IDIF complex = 0.11 μM). After incubation, the samples were eluted through illustra^TM^ NAP-5 columns (GE Healthcare, USA) and radioactivity corresponding to the collected fractions of free [^131^I]IDIF and TTR-[^131^I]IDIF complex were compared to determine the proportion of [^131^I]IDIF free and bound to TTR. For concentrations over 50 μM of IDIF, the displacement was > 97%. Hence, the [^131^I]TTR-IDIF complex was prepared as follows: the three fractions with highest radiactivity, 540 µL, were incubated with 10 µL of 3.5 mM IDIF meglumine salt solution (final IDIF concentration 63.6 µM) for 16 h at 4 °C.

### Animals experiments: general aspects

Male mice weighting c.a. 22 g (BALB/cJRj, 9 weeks, Janvier; see below for number of animals) were used. The animals were maintained and handled in accordance with the Guidelines for Accommodation and Care of Animals (European Convention for the Protection of Vertebrate Animals Used for Experimental and Other Scientific Purposes) and internal guidelines. All experimental procedures were approved by the Ethical Committee of CIC biomaGUNE and by local authorities (Diputación Foral de Guipúzcoa) before conducting experimental work.

### *Ex vivo* biodistribution studies

Animals (n = 3 per compound and time point) were anesthetized with isoflurane 5% in pure oxygen and a solution containing [^131^I]TTR, [^131^I]IDIF, [^131^I]TTR-IDIF complex or TTR-[^131^I]IDIF complex (0.9 ± 0.4 MBq/120 μl) was injected through one of the lateral tail veins. Animals were recovered from anaesthesia and at pre-determined time points (t = 5, and 15 min and 1, 6 and 24 h), animals were sacrificed by perfusion using saline solution and kidneys, spleen, liver, small intestine, and brain were quickly removed and rinsed with water. The amount of activity in each organ was measured in an automatic gamma counter (2470 Wizard, PerkinElmer). Urine and blood samples were obtained just before perfusion. Part of the blood was processed to separate the plasma, which was also counted in the gamma counter.

### Pharmacokinetic analysis of [^131^I]TTR and [^131^I]TTR-IDIF

Animals (n = 4 per compound) were administered with the labeled compounds. Blood samples (5 µL) were withdrawn at different time points using a capillary tube, and the amount of radioactivity was determined using a gamma counter (2470 Wizard, PerkinElmer). The concentration in plasma was calculated from the concentration of radioactivity in blood and the plasma-to-blood ratios determined in biodistribution experiments.

The plasma concentration versus time data were analysed by a compartmental method. The data analysis was performed by non-linear regression using the SOLVER function of the spreadsheet programme Microsoft Excel. In order to describe the disposition after the intravenous injection, two kinetic models were compared. First, the one-compartment model with bolus input and first-order elimination rate described by Eq. 1:

1 $$C(t)=\frac{D}{V}\cdot {e}^{-{K}_{el}\cdot t}$$

where *D* is the dose administered by iv injection; *K*_*el*_ is the elimination constant; and *V* is the volume of distribution in the compartment. Apparent terminal half-life *(t*_*1/2*_) is calculated as Ln2/*K*_*el*_ and the plasma clearance (Cl) for each compound is estimated as the ratio of dose/AUC (area under the plasma concentration-time curve).

The second model was a two-compartment model with bolus input and first-order elimination rate, and was described by the equation:

2 $$C(t)=D(A\cdot {e}^{-\alpha \cdot t}+B\cdot {e}^{-\beta \cdot t})$$

where *D* is the dose administered by iv injection; *α* and *β* are constants that depend solely on *K*_12_ and *K*_21_ (transfer constants between compartments 1 and 2) and *K*_10_ (elimination constant). Apparent terminal half-life *(t*_*1/2*_) is calculated as Ln2/*β* and the plasma clearance (Cl) for each compound is estimated as the ratio of dose/AUC (area under the plasma concentration-time curve).

Blood clearance (Cl_blood_) was estimated from plasma clearance (Cl_plasma_) using the following relation: Cl_blood_ = Cl_plasma_/BP, where *BP* is the blood-to-plasma ratio.

The initial estimates of the pharmacokinetic parameters were computed using curve stripping. The pharmacokinetic parameters were *V*_c_ and *K*_el_ in the case of one-compartment model. In the case of two-compartment model, the estimated pharmacokinetic parameters were *A, B, α* and *β*. From these parameters, several derived pharmacokinetic parameters were computed: AUC (*D/V·K*_*el*_ and *A/α* + *B/β* for one- and two-compartmental model. respectively), Cl (*D/AUC*), V_ss_, C_max_ (*D/V* and *A* + *B* for one- and two-compartmental model, respectively), and apparent terminal half-life. As plasma concentrations often span a wide range, it is useful to employ a weighting procedure for the raw data that allows one to fit low concentrations and high concentrations simultaneously. The choice of the appropriate weighting scheme (relative or Poisson) was performed according to the GrapPhad Curve Fitting Guide.

The Akaike information criterion (AIC) was used to identify the “best” model. This is a measure of goodness of fit and is calculated according to Eq. 3:

3 $$AIC=N\cdot \,\log (WRSS)+2\cdot P$$

The AIC considers both the model fit (sum of squared residuals) and the number of parameters of the model. N is the number of observations. WRSS is the weighted residual sum of squares, an estimate of the variance of the residuals. P is the number of parameters in the model. When comparing several models for the same dataset, the model associated with the smallest AIC value is regarded as the “best” model.

### Autoradiography studies

Animals (n = 3 per time point) were anesthetized with isoflurane 5% in pure oxygen and a solution containing [^131^I]TTR (0.9 ± 0.2 MBq/120 μl) was injected through one of the lateral tail veins. Animals were recovered from anaesthesia and at pre-determined time points (t = 15 min and 1 and 6 h), animals were sacrificed by perfusion using saline solution, the brains were harvested and promptly flash-frozen in isopentane at −50 ± 10 °C and then stored at −80 °C. Brains were sliced starting from interaural 6.60 mm and bregma 2.80 mm in a cryostatic microtome (Leica CM3050 S, Germany). The slices (20 μm thick) were disposed on glass slides. Autoradiography was performed in Beta Imager 2000 system (Biospace Lab, France), for 24 h or until more than 3 million counts were reached. Three consecutive slices at Bregma = −0.94 mm were quantified. With that aim, regions of interest were delineated manually on the photographs of the slices and translated to the autoradiography images using the M3 Vision software (Biospace Lab, Paris, France). The concentration of radioactivity in the different regions and the whole slice were obtained as cpm/mm^2^. Values were expressed as the ratio between the concentration of radioactivity in each region and the average concentration of radioactivity in the whole slice.

### *In vivo* PET studies

PET experiments were performed using an eXploreVista-CT small animal PET-CT system (GE Healthcare) to obtain complete time-activity-curves during the 80 minutes after intravenous administration. Anesthesia was induced with 3% isoflurane and maintained by 1.5–2% of isoflurane in 100% O_2_. During imaging, mice were kept normothermic using a heating blanket (Homeothermic Blanket Control Unit; Bruker). To perform the studies, [^124^I]TTR (0.44 ± 0.07 MBq, 100 μL) was injected *via* one of the lateral tail veins concomitantly with the start of a PET dynamic acquisition (energy window: 400–700 KeV). Complementary PET static acquisitions were carried out at 6 h and 24 h after administration. In all cases, two beds were defined to acquire whole body images. After each PET acquisition, a CT scan (X-Ray energy: 40 kV, intensity: 140 µA) was performed for a later attenuation correction application in the image reconstruction. Random and scatter corrections were also applied to the reconstructed images (2DOSEM iterative algorithm, 4 iterations), generating a 175 × 175 × 116 dimension image, with a 2 mm axial FWHM spatial resolution in the centre of the Field Of View (FOV).

PET-CT images of the same animal were co-registered and analyzed using PMOD image processing tool, and analyzed by visual inspection.

### Statistical analysis

For dissection and gamma counting experiments to assess biodistribution, values obtained for different compounds at each time point and organ were compared using t-student test, comparison of two independent means, using the GraphPad Prism software (version 7.03).

### Safety

The work described here comprises the manipulation of radioactive materials. All experiments were conducted in an authorized radioactive facility, and samples were handled by fully trained, accredited personnel following EU and National standards and regulations.
